# The Canadian Mother-Child Cohort Active Surveillance Initiative (CAMCCO): Comparisons between Quebec, Manitoba, Saskatchewan, and Alberta

**DOI:** 10.1371/journal.pone.0274355

**Published:** 2022-09-20

**Authors:** Anick Bérard, Padma Kaul, Sherif Eltonsy, Brandace Winquist, Dan Chateau, Steven Hawken, Ann Sprague, Mark Walker, Sasha Bernatsky, Michal Abrahamowicz, Cristiano Soares de Moura, Évelyne Vinet, Bruce Carleton, Gillian Hanley, Tim Oberlander, Odile Sheehy, Yessica Haydee Gomez, Jessica Gorgui, Anamaria Savu

**Affiliations:** 1 Research Center, CHU Sainte-Justine, Montreal, Quebec, Canada; 2 Faculty of Pharmacy, University of Montreal, Montreal, Quebec, Canada; 3 Faculty of Medicine, Université Claude Bernard Lyon 1, Lyon, France; 4 Faculty of Medicine and Dentistry, University of Alberta, Edmonton, Alberta, Canada; 5 Canadian VIGOUR Center, University of Alberta, Edmonton, Alberta, Canada; 6 College of Pharmacy, Rady Faculty of Health Sciences, University of Manitoba, Winnipeg, Manitoba, Canada; 7 Community Health and Epidemiology, College of Medicine, University of Saskatchewan, Saskatoon, Canada; 8 Research School of Population Health, Australian National University College of Health and Medicine, Canberra, Australia; 9 Clinical Epidemiology Program, Ottawa Hospital Research Institute, Ottawa, Ontario, Canada; 10 Institute of Clinical Evaluative Sciences, uOttawa Site, Ottawa, Ontario, Canada; 11 School of Epidemiology, Public Health and Preventive Medicine, University of Ottawa, Ottawa, Ontario, Canada; 12 Better Outcomes Registry & Network (BORN) Ontario, Ottawa, Ontario, Canada; 13 Department of Obstetrics, Gynecology and Newborn Care, The Ottawa Hospital, Ottawa, Ontario, Canada; 14 Divisions of Rheumatology and Clinical Epidemiology, McGill University Health Centre, Canada; 15 Department of Epidemiology and Biostatistics, School of Global & Population Health, McGill University, Montreal, Quebec, Canada; 16 Faculty of Medicine, Department of Clinical Epidemiology, McGill University, Montréal, Québec, Canada; 17 Division of Translational Therapeutics Department of Pediatrics, Faculty of Medicine, University of British Columbia, Vancouver, Canada; 18 Department of Obstetrics & Gynaecology, University of British Columbia, Vancouver, British Columbia, Canada; 19 University of British Columbia, School of Population and Public Health, Department of Pediatrics, Vancouver, British Columbia, Canada; Dalhousie University, CANADA

## Abstract

**Background:**

Given that pregnant women taking medications are excluded from clinical trials, real-world evidence is essential. We aimed to build a Canadian Mother-Child Cohort Active Surveillance Initiative (CAMCCO) and compare frequency of prematurity, low-birth-weight (LBW), major malformations, multiplicity, and gestational medication use across four provinces.

**Methods:**

CAMCCO is a collaborative research infrastructure that uses real-world data from large provincial health care databases in Canada; developed with standardized methods to similarly construct population-based pregnancy/child cohorts with longitudinal follow-up by linking administrative/hospital/birth databases. CAMCCO also includes a common repository to i) share algorithms and case definitions based on diagnostic and procedural codes for research/training purpose, and ii) download aggregate data relevant to primary care providers, researchers, and decision makers. For this study, data from Quebec (1998–2015), Manitoba (1995–2019), Saskatchewan (1996–2020), and Alberta (2005–2018) are compared (Chi-square tests, p-values), and trends are calculated using Cochran-Armitage trend tests.

**Results:**

Almost two-thirds (61%) of women took medications during pregnancy, mostly antibiotics (26%), asthma drugs (8%), and antidepressants (4%). Differences in the prevalence of prematurity (5.9–6.8%), LBW (4.0–5.2%), and multiplicity (1.0–2.5%) were statistically significant between provinces (p<0.001). Frequency of major malformations increased over time in Quebec (7–11%; p<0.001), Saskatchewan (5–11%; p<0.001), and Alberta (from 7–8%; p<0.001), and decreased in Manitoba (5–3%; p<0.001). Cardiovascular and musculoskeletal malformations were the most prevalent.

**Interpretation:**

Medications are often used among Canadian pregnancies but adverse pregnancy outcomes vary across provinces. Digitized health data may help researchers and care providers understand the risk-benefit ratios related to gestational medication use, as well as province-specific trends.

## Introduction

In recent years, large national administrative databases or registries have been increasingly used in the field of perinatal pharmacoepidemiology, recognizing the importance of large size longitudinal pregnancy and child cohorts, and the need for and value of harmonizing distinct datasets [1–4]. This is also due to the fact that at least 50% of pregnant women take medications [5] for which very few safety and effectiveness data exists given that these women are systematically excluded from most clinical trials before a drug is approved for post-marketing use. Therefore, in this specific patient population, healthcare administrative, hospital, and sociodemographic databases have become a cornerstone in the process of assessing performance and providing feedback to improve quality of health care delivery and outcomes at a population-level. They provide real world data (RWD) leading to real world evidence (RWE), and allow a broader range of questions to be answered.

Healthcare in Canada, which theoretically provides universal access to physician services and hospital care, is mostly administered by provinces. This results in the accumulation of a wealth of data annually representing real-life use of services, diagnoses, hospitalisations and medication use. Collectively, these data provide insights into the health of the Canadian population that can be used for policy making and guidelines to increase wellbeing and decrease risk. Availability of digital health data provides important opportunities for outcomes research, including comparisons between jurisdictions. Routinely collected health care data also allows for the detection of infrequent events, as well as long-term infrequent outcomes. This is important since very little research on fetal drug exposure and associated outcomes goes beyond the first year after birth. Building new pregnancy and child cohorts using existing health care data takes many months or even years because a number of databases need to be linked and variables need to operationalized, coded, and harmonized in order to give valid answers. This lengthy process can draw out, and often prevent, valuable research. Having standardized cohorts ready to be used will expedite investigations, and allow for near real-time queries that have the potential for greater impact on patient care.

Although health data are routinely collected in all Canadian provinces, they were not primarily intended (or validated) for research purposes. Up until recently, Canada did not have a standardized and harmonized infrastructure to perform state-of-the-art research in perinatal epidemiology. Although the Canadian Perinatal Surveillance System [6] is ongoing, it does not allow for near real-time analyses, nor provide harmonized common data models including drug data. Leveraging funding from Health Canada and innovation funding from the Canadian Foundation for Innovation, we have established and built an efficient organizational infrastructure to develop the Canadian Mother-Child Cohort Active Surveillance Initiative (CAMCCO). The ultimate goal of CAMCCO is to identify serious events associated with gestational medication use in a timely manner, which allows us to take preventive measures in order to avert or minimize them. An equally important benefit of CAMCCO is that it can be used as the foundation of a range of perinatal, child, and maternal health studies, and allow for long term follow-up, into adulthood (lifespan approach). Indeed, the added-value of CAMCCO is that it provides fast-track real-time access to harmonized pregnancy-mother-child provincial cohorts including data on medication fillings in pregnancy and childhood as well as clinical, hospital and socio-demographic data; CAMCCO can also be used as a training platform, which will lead to an enduring knowledge-transfer strategy. CAMCCO also has value in leveraging RWD to study differences in prescribing practices and medication use during pregnancy and childhood, and quantify the impact of gestational medication use on perinatal outcomes across Canada. CAMCCO is a sentinel system for safety signals related to drug exposures during pregnancy in Canada.

CAMCCO has put together a multidisciplinary team across 6 provinces (the remaining 4 Maritimes provinces will be included soon) to harness the power and value of RWD present in population-based provincial health databases. The aim of this current manuscript is to present CAMCCO and compare descriptive baseline rates of gestational medication use, prematurity, low birth weight (LBW), multiplicity, and major congenital malformations between Quebec, Manitoba, Saskatchewan, and Alberta.

## Methods

The CAMCCO infrastructure leverages provincial databases of physician visit, hospital and sociodemographic RWD. This is done in order to put in place harmonized provincial mother-child cohorts with longitudinal follow-up of mothers and children from 1998–2021 (updated quarterly) that is done with the development and use of standardized and harmonized diagnosis and medication codes, programming coding, SAS algorithms, common data model (which enables the capture of similar datasets with the same variables across provinces) that enables database linkages, follow-up, and identification of variables (beginning and end of pregnancy, trimester definition, medication exposure algorithms, mother-child link, major malformations, prematurity, LBW, etc) in a similar manner across provinces. CAMCCO is the underlying infrastructure on which a state-of-the-art research program will be established with common protocols at the provincial level et meta-analyses methodology at the Canadian level. This is done for ultimately generating aggregate measures (annual means, risks and prevalence measures) in the CAMCCO secure repository (Canadian repository (www.motherchildcohort.ca)), which will be used for perinatal surveillance (S1 Fig). The CAMCCO secure website is also used for sharing of algorithms and programming codes among team members during collaborative projects across provinces for harmonization purpose (S1 Fig). At present, CAMCCO includes approximately 4 million Canadian pregnancies and 3 million children with up to 23 years of follow-up (1998–2021). CAMCCO is one of the largest and most representative longitudinal cohorts of pregnancies, mothers and children in the world. It includes harmonized provincial cohorts in Alberta, Saskatchewan, Manitoba, and Quebec. The British Columbia, and Ontario Mother-Child cohorts will begin development shortly; the remaining 4 provinces will also be included in CAMCCO soon.

### Databases *already* available to researchers in each province (S1 Table)

The medical service databases (Medical Services file) contain detailed information on maternal age, welfare status, place of residence (urban vs. rural); and all medical services, including medical and emergency department visits, hospitalisations, diagnoses and procedures performed by physicians and nurse practitioners. Health care provider characteristics are also included (e.g. specialty, year of graduation). The prescription drug file covers information on all outpatient filled prescribed medications, the prescribing physician and dispensing pharmacist, drug name, dosage, formulation, quantity dispensed, date or duration of the dispensation, except for Quebec who only has data on filled medications prescribed to those insured by its public drug plan. For all provinces, over-the-counter (OTC) medications that are not prescribed and filled are not accounted for. Furthermore, medication data on dosage is not available in Saskatchewan. Data in the prescription drug files have been validated in the Quebec Mother-Child cohort, and found to be highly reliable [7]. The hospitalization databases (Discharge Abstract Database and National Ambulatory Care Reporting System) record all acute care hospitalizations, day surgery, outpatient and emergency visits. Up to 25 discrete diagnoses are coded by trained health information professionals and include physician-based medical diagnoses. CAMCCO’s algorithm used to define gestational age has been validated in the Quebec Mother-Child Cohort and compared against medical charts [8]. Birth and death databases provide demographic information on the mother, father, and baby such as marital status, and birth weight and gestational age for live births and stillbirths and cause of death. Vital data registries have been compared to medical charts and found to be complete and valid.^8^ Although these databases provide a wealth of RWD, they were not readily available for research and hence required extensive manipulation–therefore CAMCCO developed standardized and harmonized codings and processes across provinces.

### Development of province-specific cohorts

In order to create the province-specific cohorts, linkage between the already existing administrative, hospital and socio-demographic databases listed above is done using a patient unique encrypted identifier linking mothers to babies (S2 Fig). Pregnant women are identified in the databases by a prenatal visit or by a therapeutic procedure related to pregnancy (e.g., ultrasound, amniocentesis, etc.). Women are followed from the beginning of pregnancy, defined as the first day of the last menstrual period, until the end of pregnancy (planned or spontaneous abortion, or delivery), which is defined using data on gestational age, which has been validated against patient-charts [8] (S3 Fig). The status of the newborn is obtained via the births and deaths database or by linking the mother-child hospital patient charts. Given the status of the available databases linked within CAMCCO, women are treated and followed prospectively as part of the usual health care management during and after pregnancy, and children are similarly followed after birth. Anonymized individual data cannot leave the province. However, all provincial analyses are being performed within each province using the individual crude data. The crude cohort data for QC are housed at CHU Ste-Justine (University of Montreal); ON, Institute for Clinical Evaluative Sciences Ottawa (University of Ottawa); MB, Manitoba Center for Health Policy (University of Manitoba); SK, Saskatchewan Health Quality Council (University of Saskatchewan); AB, VIGOR Center (University of Alberta); and BC, at UBC. CAMCCO’s repository of aggregate data is housed at CHU Ste-Justine, QC.

In order for a pregnancy to be included in CAMCCO, continuous medication data coverage during pregnancy has to be available in the provincial medical services databases. This criterion does not impact Mother-Child cohort selection in Manitoba, Saskatchewan and Alberta, who capture all medication fillings. However, it has an impact on the Quebec Mother-Child cohort where approximately 30% of Quebec pregnant women are insured by the public medication insurance, and thus provide data to the medical services database. Nevertheless, Bérard and Lacasse^9^ have shown that pregnant women insured by the public medication insurance had similar comorbidities than those insured by private drug insurance plans in Quebec; they are however of lower sociodemographic status [9,10].

A woman can have more than one pregnancy during follow-up. Given the nature of the data that we have, no losses-to-follow-up were observed unless women moved out of the province, which did not happen during any studied pregnancies. Given that physicians, pharmacist and hospitals are paid for each service or medication dispensed, missing data is not a concern.

### Baseline indicators

Although many indicators are of interest in relation with gestational medication use, the following were used as a starting point. Others will be added to CAMCCO in the near future.

Malformations (according to the EUROCAT classification using ICD9 or ICD10 codes [11], and Blais et al. [12]) are identified in the first year of life; major malformations are combined and also categorized by organ systems (S2 Table). Twelve months after delivery is necessary to identify major malformations to take into account late reportings as well as validate early diagnoses. Quebec and Alberta record all malformations regardless of count size whereas Manitoba do not report categories with less than 5 counts, which has an impact on the calculation of rates.

LBW (calculated using data on birth weight from birth records (<2500 g), and typically excludes stillbirths) is defined at birth; and prematurity (using data on gestational age, <37 weeks gestation; extremely preterm <28 weeks gestation, very preterm 28–32 weeks gestation, moderate to late preterm >32-<37 weeks gestation) is defined at delivery. Multiplicity is defined as having delivered more than one newborn (vs. singleton).

All indicators are harmonized across province as well as with other international registries [2,3,11]. They are counted annually, and annual rates per province are then calculated.

Medication exposures are categorized using the drug identification numbers (DIN); exposure during the first trimester is defined as anytime between the first 14 weeks of gestation (from the first day of the last menstrual period), the second trimester between 15–26 weeks gestation, and the third trimester as exposure after the 26^th^ weeks gestation until end of pregnancy.

For this study, baseline data from Quebec (1998–2015), Manitoba (1995–2019), Saskatchewan (1996–2020), and Alberta (2005–2018) are compared (Chi-square tests, p-values), and trends were calculated using the Mann-Whitney trend test. Differences in the number of years available that could be analyzed are due to the validity of the data within each province.

### CAMCCO repository

Finally, the province-specific individual record-level data remain in the province but provincial aggregate indicators are downloaded in a secure repository in Montreal–CHU Ste-Justine (www.motherchildcohort.ca) (S1 Fig). Other aggregate measures that help to monitor determinants and outcomes of maternal, fetal, infant and child health will be generated as part of future research using CAMCCO RWE–these will also be downloaded in the repository as they are produced. This will allow fast-track assessment of health indicators across provinces in order to plan clinical and maternal relevant research, aid policy makers, identify signals, or determine feasibility of international collaborations on specific relevant topics. This will also provide real-time active surveillance on the prevalence and impact of medication use during pregnancy. In addition, this will allow for state-of-the art research to be performed with high validity in a timely manner in order to have significant impact on health decisions and patient care. The databases are updated in real-time given that they are part of the usual health care system in Canada. For active surveillance purposes, the CAMCCO cohort updates rates annually in its repository.

The secure CAMCCO website is also used to share, circulate, and harmonize codes and important standardized methodology (definitions, algorithms, and programming) within the team members and programmers (S1 Fig). The aim is that the CAMCCO repository eventually becomes Open Source to further accelerate perinatal pharmacoepidemiology research.

## External data access

Centralization of external data access requests and analyses are done to ensure the validity and reproducibility of findings. Everyone requesting data access and doing statistical analyses are signing a confidentiality agreement form. All requests need to include at least one CAMCCO investigator to ensure harmonization of reporting. Access requests to CAMCCO for research purposes is done to A Bérard, and approved by the Steering Committee and provincial ethics committees. In addition, there are various groups that could benefit from using CAMCCO for purposes other than drug effects research; decision making, public health, etc. A more flexible process is thus being developed that would enable timely access for non-research purposes (eg. running pregnancy rates per health network). Hence, processes based on goodwill between CAMCCO’s provincial leads and provincial data partners who need to approve such requests for non-research purposes are being done locally.

## Ethics approval

This study was approved by the Quebec Data Access Agency and the CHU Sainte-Justine Institutional Review Board (#1740 and #2976). Additionally, ethics approval by province was obtained for Saskatchewan, Manitoba and Alberta. All data were analyzed anonymously using provincial data granted to us.

## Data sharing

Given that underlying data come from third parties, there are restrictions on publicly sharing data. Hence, such data cannot be made available. In order to obtain crude underlying health data from billing, hospital and birth certificate databases in each of the four provinces, individuals need to contact data holders of the 3 databases within each specific province (Quebec, Manitoba, Saskatchewan, Alberta). Aggregate data presented in this manuscript (prevalences, means, etc.) are available to readers.

Institutional contacts for data access: 1) in QC, CHU Ste-Justine Ethics Committee (http://chusj.nagano.ca), Montreal, QC, Canada for researchers who meet the criteria for access to confidential data; 2) in MB, University of Manitoba and MCHP Institutional Data Access / Ethics Committee (https://umanitoba.ca/manitoba-centre-for-health-policy/) for researchers who meet the criteria for access to confidential data; 3) in SK, Saskatchewan Health Quality Council (www.saskhealthquality.ca); 4) in AB, University of Alberta Ethics Committee, (www.ualberta.ca/research/research-support/research-ethics-office/human-research-ethics/research-ethics-boards/).

## Results

For this manuscript, surveillance results on key perinatal indicators from Quebec, Saskatchewan, Manitoba, and Alberta are presented. S4 Fig presents the calendar time period on which these preliminary results have been generated; for the four provinces, 25 years of data are available, and across province overlap is present from 2005 to 2015. The Alberta Mother-Child cohort is the largest (688,270), followed by Manitoba (347,888), Saskatchewan (291,324), and Quebec (248,787).

Seventy-two percent (72%) of women took medications in the year before pregnancy. This decreased to 61% during pregnancy and increased to 79% in the year after pregnancy; the majority of exposures (89%) occur during the first trimester, organogenesis. The majority of medications used during pregnancy were antibiotics (26%) to treat infections, anti-emetics (14%) to treat nausea and vomiting of pregnancy, asthma drugs (8%), and antidepressants (4%) for depression, anxiety and pain; high dose folic acid was used in 7% of pregnancies. No differences were observed between provinces.

**Fig 1:**
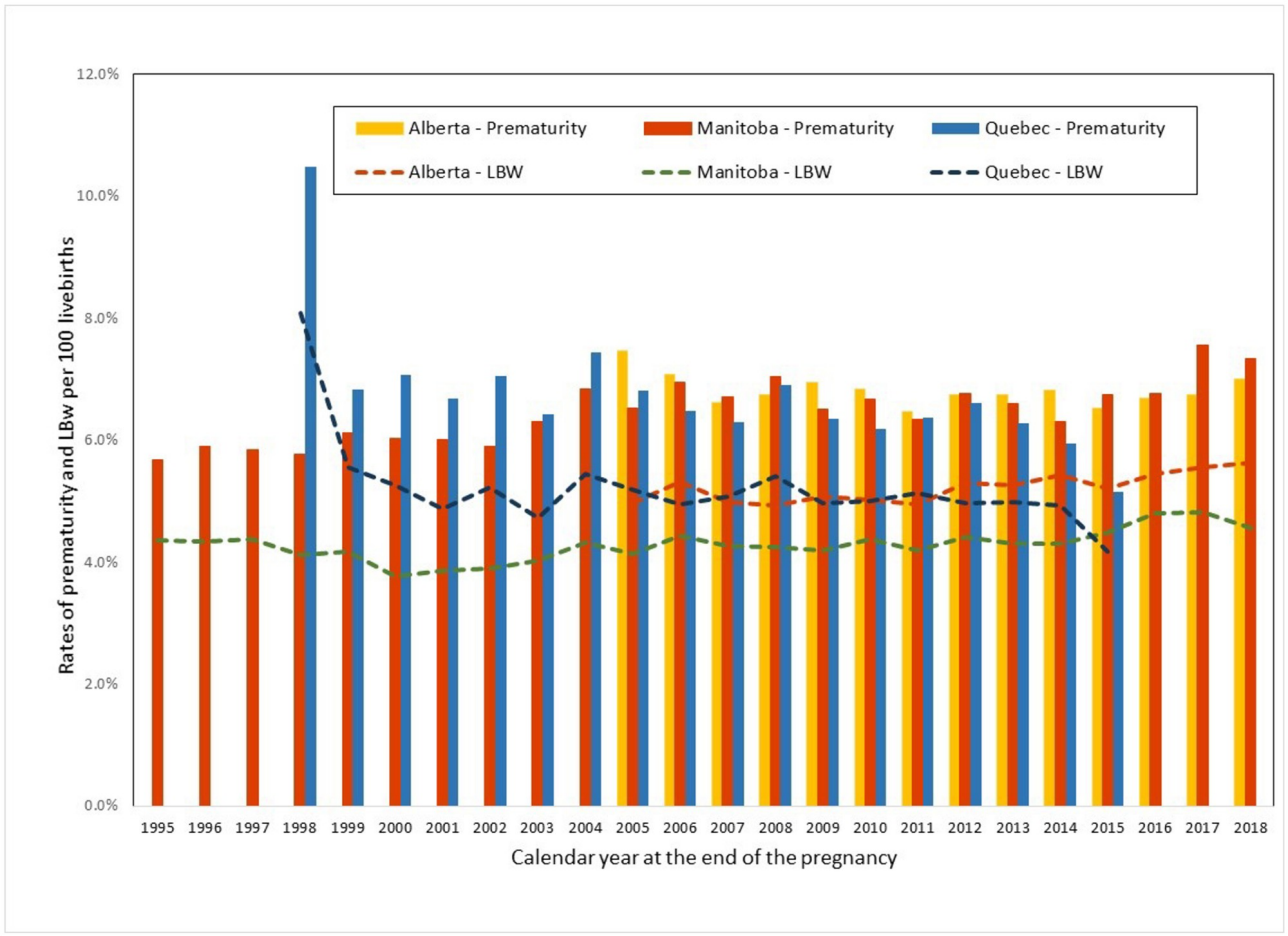
CAMCCO—Quebec, Manitoba, Saskatchewan, and Alberta—Annual rates of prematurity and low birth weight (LBW). Annual rates of prematurity and LBW during the study period in Quebec, Manitoba, Saskatchewan, and Alberta. Note: Data on preterm births were missing from SK’s birth abstracts for the years 1996–2000; and birthweight was missing for the years 1996–98 for live births.

**Fig 2:**
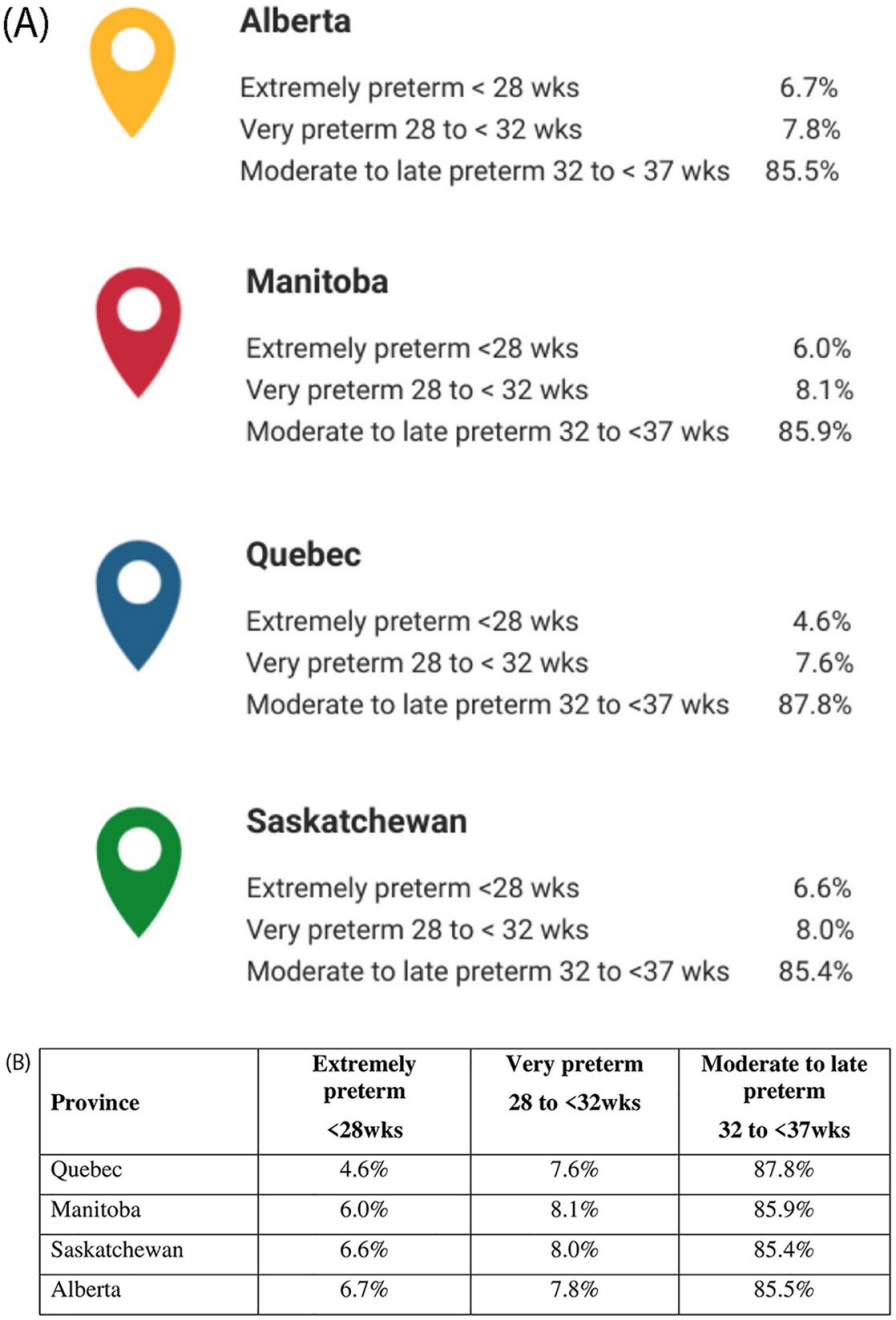
CAMCCO—Quebec, Manitoba, Saskatchewan, and Alberta—Overall prevalence of categories of prematurity. Note: Data on preterm births were missing from SK’s birth abstracts for the years 1996–2000.

Slight variations in the annual rates of prematurity and LBW from 1995–2020 were observed between provinces (Fig 1) (mostly due to incompleteness in reporting during the first calendar years of CAMCCO data), but provincial time trends were constant over time (p>0.05). Differences in the overall prevalence of prematurity, LBW, and multiplicity were statistically significant between provinces (p<0.001) but not clinically significant (S5 Fig). The majority of premature births were moderate to late preterm (32–37 weeks gestation) (86%), and similar findings were obtained across provinces when stratifying on categories of prematurity (Fig 2).

**Fig 3:**
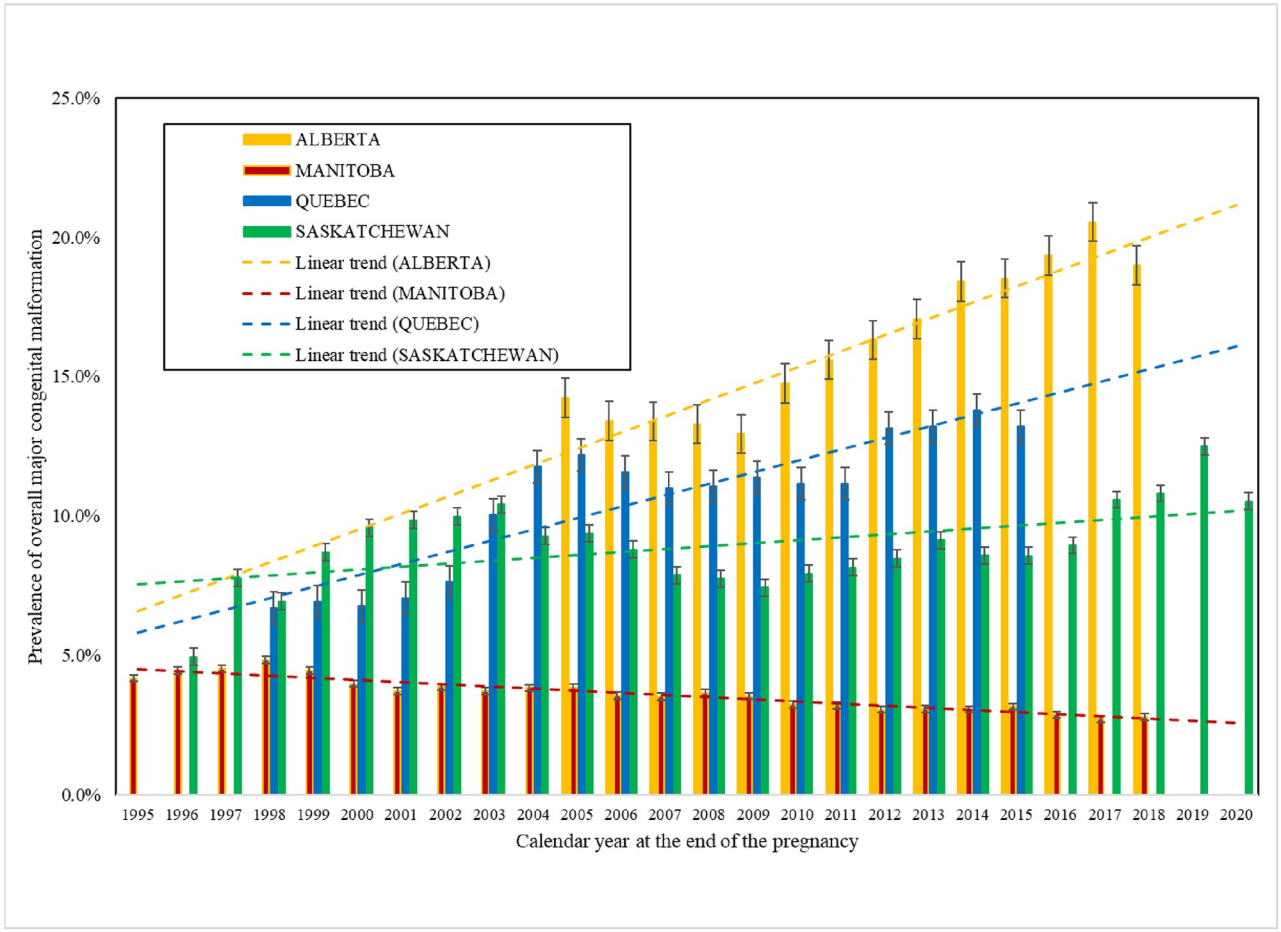
CAMCCO—Quebec, Manitoba, Saskatchewan, and Alberta—Annual rates of major malformations in the first year of life with trends for each province. Note: Trend comparisons between provinces p < 0.0001.

Annual rates of major malformations in Quebec, Manitoba, and Alberta are presented in Fig 3. Between-province variations in the rates of major malformations were observed. Alberta and Quebec systematically reported higher risk of malformations, with Quebec having the highest prevalence. Overall, the prevalence of major malformations increased over time in Quebec (7–11%; p<0.001), in Saskatchewan (5–11%; p<0.001), and in Alberta (from 7–8%; p<0.001), and decreased in Manitoba (5–3%; p<0.001).

**Fig 4:**
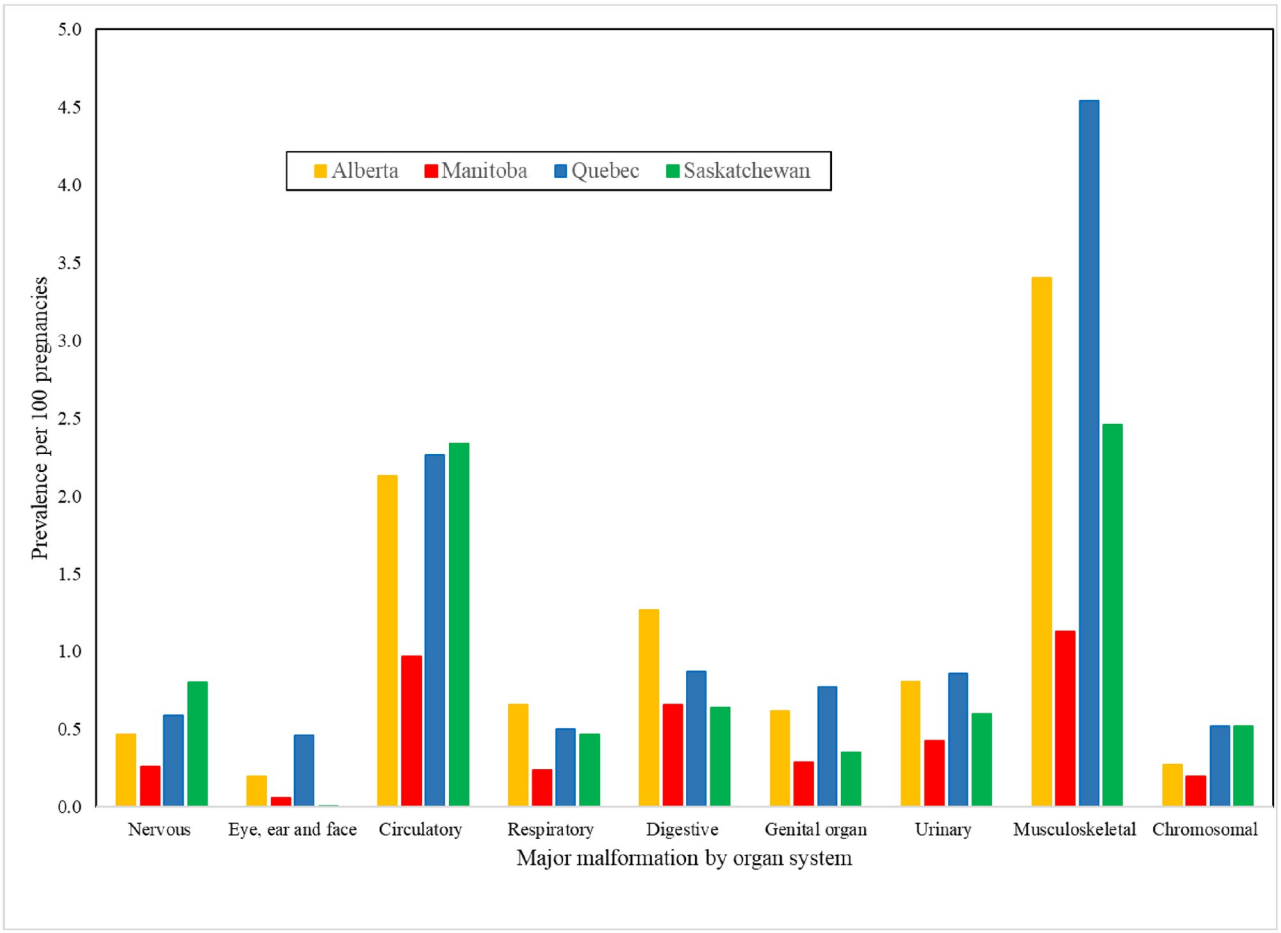
CAMCCO—Quebec, Manitoba, Saskatchewan and Alberta—Prevalence of major malformations in the first year of life by organ system. Note: All major malformation organ system prevalences between provinces were statistically significant (p < 0.0001).

Circulatory system malformations, which include heart defects, and musculoskeletal malformations are the most prevalent (Fig 4). Further looking at specific malformations, cardiac defects were the most prevalent (1.91% QC, 0.76% MB, 0.76 SK, 1.72% AB); ventricular and atrial septal defects were the most prevalent cardiac defects identified (S6 Fig). Alberta reported the highest prevalence of pyloric stenosis, gastroschisis, cleft lip with or without palate, and omphalocele whereas Quebec reported the highest prevalence of ventricular and atrial septal defects (S6 Fig).

## Discussion

CAMCCO is the first harmonized Mother-Child Cohort infrastructure in Canada, which provides RWD on medication exposure during pregnancy. In addition of being population-based, it includes up to 22 years of follow-up for mothers and children, allowing CAMCCO to study short- and long-term impact of gestational medication exposure. Furthermore, the CAMCCO repository is being used as an active surveillance tool to assess trends in gestational medication use as well as adverse pregnancy outcomes across Canada.

These first CAMCCO baseline analyses have shown that although Quebec is more populated than Manitoba, Saskatchewan, and Alberta [13], it provided fewer pregnancies and children data to CAMCCO, which can be explained by the fact that we did not have access to all the pregnancy data in the province. Indeed, Bérard and Lacasse [9] have shown that 30% of pregnant women are insured by the Quebec public medication insurance program. Although pregnant women insured by private and public medication plans have comparable comorbidity profiles,^9^ it remains that they could have different gestational medication use patterns, which could be a limitation for the Quebec Mother-Child cohort.

Although medication exposures decreased during pregnancy, 61% of pregnant women were using medications during the gestational period, mostly during organogenesis (89%). This is consistent with the literature [2,3,11,14] and the fact that 50% of pregnancies are unplanned, increasing the likelihood of inadvertent drug exposures [5]. The most frequent medications were antibiotics, anti-emetics, asthma drugs, and antidepressants, which is consistent with Bérard et al. [10] and Haas et al [15]. Although not clinically significant, there were differences between provinces in the annual rates of prematurity, and LBW with Saskatchewan having the lowest frequency, and Alberta the highest. However, Manitoba had the highest prevalence of multiplicity compared to the other provinces. Alberta and Quebec systematically reported higher annual rates of malformations, 16.4% and 10.4%, respectively. This is consistent with Quebec’s founders’ effect [16] (i.e. a relatively small number of French settlers were ancestors of a large number of families in France), and Zhao et al. [17]; it could also be explained in part by the fact that women of lower socio-economic status have poorer health or poor lifestyle habits. Alberta’s prevalence of major malformation of 16% is higher than the 2013 Public Health Agency of Canada survey [18], which showed a 6.25% baseline prevalence of malformations in this province. The prevalence of major malformations observed in Saskatchewan is 9%, which is close to the one observed in Quebec. Manitoba had rates between 3–5%, which is consistent with other countries [2,3,11,14]. The rates of major malformations increased over time in Quebec, Saskatchewan, and Alberta, and decreased in Manitoba, which could be explained by the fact that cells with counts lower than 5 are not reported in Manitoba, hence underreporting cases. Although variations in prevalences were observed, cardiac defects, and musculoskeletal malformations were the most prevalent in the four provinces, which is consistent with the literature [2,3,11,14]. Alberta reported the highest prevalence of pyloric stenosis, and omphalocele whereas Quebec reported the highest prevalence of ventricular, and atrial septal defects. For its part, Saskatchewan reported the highest prevalence of cleft lip with or without palate, and Manitoba the highest prevalence of gastroschisis. Variations between provinces could further be explained by differences in race and ethncity, culture with regards to having children (Quebec has lower birth rates), proportions of First Nations and Metis (Manitoba, Saskatchewan, and Alberta have the largest proportions of First Nations and Metis, who often have relatively high number of pregancies).

Given the surveillance nature of the presented CAMCCO data in this manuscript, we cannot imply an association between medication exposure during pregnancy and the presented health indicators including medication exposure. The purpose here was to present the CAMCCO infrastructure and methodology, which can be used for real-time etiologic research, training, and knowledge transfer, and compare provincial health indicator rates between provinces.

The fact that the first four CAMCCO provincial cohorts have produced comparable rates on prematurity, LBW and multiplicity shows reliability in the harmonization procedures; these prevalences are also comparable to estimates from Statistics Canada [19], which increases the validity of the four CAMCCO cohorts. There were variations on rates and types of major malformations between provinces but these were consistent with other estimates already published [2,3,11,14]. This shows that CAMCCO is a reliable Mother-Child infrastructure to perform research and obtain evidence-based findings.

CAMCCO has potential limitations including the fact that over-the-counter medications and in-hospital dispensed medications are not accounted for. This could potentially underestimate medication exposure before, during and after pregnancy, as well as in children. Data on medications are based on prescription fillings, which could not necessarily represent actual intake. However, Zhao et al. [7] have shown high positive and negative predictive values of data in our databases compared to real-time maternal report.

CAMCCO is the first infrastructure of its kind that leverages RWE to quantify the benefits/risks of medication use during pregnancy for mothers and children in a timely, harmonized, and valid manner in order to have a significant impact on i) how medications are used and prescribed during pregnancy and childhood, and ii) ultimately on the development of revised guidelines and implementation of revised health policies on the treatment of maternal conditions during and after gestation. With its infrastructure and repository, CAMCCO collaborates with researchers, knowledge-users, and decision makers as well as patient representatives, addressing the gaps between the lack of randomized controlled trial data on medication use in pregnant women and children.

Given the high prevalence of medication exposures during pregnancy and the lack of randomized clinical trials showing efficacy and risks during this crucial developmental period, it is of pivotal importance to determine the impact of gestational medication use on the physical and mental health of mothers and children. CAMCCO leads to fast-track, valid and reproducible RWE that will be used for decision making, and clinical practice guidelines based on state-of-the-art infrastructures. Other than being urgently needed, it has the potential of becoming one of the most powerful research infrastructures generating evidence-based findings and RWE in the world, while greatly expanding Canada’s capacity to conduct research and consolidate its role as an international leader in maternal and child health and medication use during pregnancy.

## Supporting information

S1 Table Database available in CAMCCO. (PDF)

S2 Table ICD-9 and ICD-10 diagnostic codes for major congenital malformation by organ system. (PDF)

S1 Fig CAMCCO infrastructure. (PDF)

S2 Fig CAMCCO—Linkage procedures within each province. ICD 9–10: International Classification of Diseases 9th and 10th Revisions; DIN: Drug Identification Number; PIN: Unique Personal Identification number for mothers and children within each province.
(PDF)

S3 Fig CAMCCO—Provincial cohort structure. (PDF)

S4 Fig CAMCCO—Quebec, Manitoba, Saskatchewan, and Alberta—calendar years included in this study. (PDF)

S5 Fig CAMCCO—Quebec, Manitoba, Saskatchewan, and Alberta—Overall prevalence of prematurity, LBW, and multiplicity. (PDF)

S6 Fig CAMCCO—Quebec, Manitoba, Saskatchewan, and Alberta—Prevalence of specific malformations in the first year of life. Note: All specific major malformation grouping prevalences between provinces were statistically significant (P<0.0001).
(PDF)
