# Supplementary material for: The Canadian Mother-Child Cohort Active Surveillance Initiative (CAMCCO): Comparisons between Quebec, Manitoba, Saskatchewan, and Alberta
Source: PLoS One. 2022 Sep 20;17(9):e0274355. doi: 10.1371/journal.pone.0274355 (PMC9488808; doi:10.1371/journal.pone.0274355)
Supplement: S1 Table — (PDF) [file pone.0274355.s001.pdf]

S1 Table. Databases available in CAMCCO

	Dictionary	Description	Available databases within each province	CAMCCO Repository (Aggregate Data)
**Province – Where linkage will be done**			QC, ON, MB, SK, AB, BC	
**Brief Database Description**	Description of database including origins, unique characteristics, general or specific users, as well as strengths and limitations of the database		Each province-specific Mother-Child Cohort is built by linking administrative, hospital and sociodemographic databases. It contains data on pregnant women, mothers and children (medical charts, lifestyle data, family and personal medical history) from 1995-2021. The provincial Mother-Child cohorts are updated annually (data on mothers, children, new pregnancies). These 6 Mother-Child provincial cohorts have the advantage of large size compared to the other data sources whose sample sizes are often insufficient to rule out low-to-moderate increased risks for even the more commonly occurring adverse perinatal outcomes such as heart defects, neural tube defects, and oral clefts. All linkages are done using denominalized unique identifiers for mothers/children; the mother-child link is ensured by a unique mother-baby link already available in each province.	Aggregate data (population means, prevalence, risk ratios/odds ratios/hazard ratios on all important/relevant variables) are being calculated within each province, using provincial-based Mother-Child Cohorts and are centralized within a unique repository in QC (secure server, CHU Ste-Justine). **It gives us the opportunity to answer policy relevant questions without reanalyzing all the individual cohorts.** In the event that more refined measures of risk are needed, a common protocol will be developed and analyses within each province will be done and results meta-analyzed.
**Database Type**	Description of type and scope of data: Population Database	**I. <u>Longitudinal Population Database</u>**<br>**A. Drug and Diagnosis Data**<br>Medical and Pharmacy Insurance Claims – *outpatient and inpatient*<br>**B. Diagnosis Data only**<br>1. Medical Claims<br>2. Electronic Records<br>3. Disease Specific<br>**C. Drug Data only**<br>Pharmacy-based – *medications fillings, outpatient*<br><br>**II. <u>Spontaneous Reporting System</u>**<br>**A. Standard**<br>**B. Usual Universal Care**	Longitudinal Provincial Mother-Child Population Database	
**Database Source**	Data origin: e.g., medical insurance and medication filling claims, hospital archives, demographic databases (births and deaths)		**Medical Insurance Claims:** Physician visits, diagnoses with calendar date (ICD9-10 codes), procedure codes, medication fillings (calendar date, drug identification number (DIN), dosage, duration), sociodemographic data on prescribers, visits to emergency). **Hospitalization database (hospital archives):** Diagnoses during hospitalization (calendar date), gestational age, procedures, data of delivery or end of pregnancy (spontaneous or planned abortion). **Birth and Death certificate data:** Data on child (gestational age at birth, birth weight), marital status of mother, education level, race/ethnicity.	Aggregate data from all 6 provinces: Population means, prevalence, risk rations/odds ratios/hazard ratios, etc. on all important/relevant variables.
**Frequency of Data Collection**	How often data is collected, such as ongoing, annually or quarterly		Real-time usual care collection.	All aggregated data produced as studies will be performed in the province-specific cohorts will be downloaded in CAMCCO Repository.
**Frequency of Data Update**	How often the database is updated		Provincial cohorts are updated annually.	
**Years Covered**	Which period the database covers		1995-2021	1995-2021
**Population Type**	Description of population type (by demographic, insurance status, region)	General Population<br>Outpatient/Non-institutionalized<br>Inpatients<br>Emergency department<br>Pregnant women<br>Mothers<br>Neonates<br>Children	General Population<br>Outpatient/Non-institutionalized<br>Inpatients (hospital data)<br>**Note:** QC only covers welfare recipients or adherents for their medications; ON only covers welfare recipients and families with children for their medications; BC, AB, SK, MB cover all their population for their medication. All other health visits and procedures are universally covered.	All aggregated data produced as studies will be performed in the province-specific cohorts will be downloaded in the CAMCCO Repository.
**Date of Last Update**	When the database was last updated		The QC Cohort has been developed for the period 1998-2015 for mothers/children covered by the RAMQ for their medications (30% of the overall population) – we are updating the cohort for 2015-2021. All the other 5 provinces are built, harmonized with the QC cohort for the period 1995-2021.	
**Size**			QC: At present – 500,000 pregnancies and 400,000 children followed for up to 18 years (1998-2015) – it is estimated that with the additional data requests (2015-2021) QC will have longitudinal data on 800,000 pregnancies and 650,000 children. ON – it is estimated that we will have data on 1 million pregnancies and 750,000 children. MB, SK, AB - 500,000 pregnancies and 400,000 children for each province. BC – 700,000 pregnancies and 550,000 children. Overall, approximately 4 million pregnancies and 3.15 million children.	
**BORN (ONTARIO)**				
-BORN Ontario is a prescribed registry under Ontario PHIPA (Personal Health Information Protection Act), which collects, discloses, and uses personal health information for the purpose of improving care and patient outcomes. The BIS, an Internet-based data collection system, is operational in all 96 hospitals providing maternal-newborn care and has data for all hospital births since 2006. Maternal demographics and health behaviours, pre-existing maternal health problems, obstetric complications, intrapartum interventions, and maternal and newborn outcomes are captured at the time of birth. Each site has access to their own data and BORN Ontario reports on outcomes aggregated at the provincial level at regular intervals. An ongoing data validation process assures high data quality, and several studies using BORN data have been published. With comprehensive capture of high-quality data for all births in the province, BORN developed reporting tools to alert hospitals to potential practice issues to facilitate practice improvement. The BORN database is in the process of being linked to the ON administrative, hospital, and socio-demographic databases – this will be the ON CAMCCO Mother-Child cohort.				
