# Supplementary material for: The Canadian Mother-Child Cohort Active Surveillance Initiative (CAMCCO): Comparisons between Quebec, Manitoba, Saskatchewan, and Alberta
Source: PLoS One. 2022 Sep 20;17(9):e0274355. doi: 10.1371/journal.pone.0274355 (PMC9488808; doi:10.1371/journal.pone.0274355)
Supplement: S2 Table — (PDF) [file pone.0274355.s002.pdf]

S2 Table. ICD-9 and ICD-10 diagnosis codes for major congenital malformation codes by organ system.

**Organ system**	**ICD-9 code**	**ICD-10 code**
Nervous system	740.0-742.9	Q00.0-Q07.9
Eye, ear, face and neck	743.0-744.9<br>*Exclusions*: 743.6, 743.8, 744.1- 744.9*	Q10.0-Q18.9<br>*Exclusions: Q10.0-Q10.6, Q13.0, Q13.2, Q13.5, Q15.8, Q17.0-Q17.5, Q17.8, Q17.9, Q18.0-Q18.9*
Circulatory system	745.0-747.9<br>*Exclusions: 747.0, 747.5*	Q20.0-Q28.9<br>*Exclusions: Q25.0, Q27.0*
Respiratory system	748.0-748.9<br>*Exclusions: 748.2, 748.3*	Q30.0-Q34.9<br>*Exclusions: Q30.2, Q30.8, Q31-Q32, Q33.1*
Orofacial clefts	749.0-749.2	Q35.0-Q37.9<br>*Exclusion: Q35.7*
Digestive system	750.0-750.9<br>*Exclusions: 750.0, 750.1, 750.2, 750.5, 750.6, 751.0, 751.5*	Q38.0-Q45.9<br>*Exclusions: Q38.1-Q38.6, Q40.0, Q40.1, Q43.0, Q43.4-Q43.9*
Genital organs	752.0-752.9<br>*Exclusions: 752.4, 752.5, 752.8*	Q50.0Q56.9<br>*Exclusions: Q52.2-Q52.8, Q53, Q54.4, Q55.1, Q55.2, Q55.6, Q55.8, Q55.9*
Urinary system	753.0-753.9<br>*Exclusion: 753.6*	Q60.0-Q64.9<br>*Exclusions: Q61.0, Q62.7, Q63.3, Q64.2, Q64.3*
Musculoskeletal system	754.0-756.9<br>*Exclusions: 754.0, 754.1, 754.7, 754.8, 756.0, 756.2*	Q65.0-Q79.9<br>*Exclusions: Q65.3-Q65.6, Q66.2, Q66.3, Q66.5-Q66.9, Q67.0-Q67.4, Q67.6-Q67.8, Q68.0. Q68.1, Q68.3-Q68.8, Q70.3, Q74.1, Q75.0, Q75.2, Q75.3, Q75.8, Q76.0, Q76.5, Q79.5, Q79.8*
Integument	757.0-757.9<br>*Exclusions: 757.2-757.6, 757.8*	Q80.0-Q84.9<br>*Exclusions: Q81, Q82.1- Q82.8, Q83.2, Q83.3, Q83.8, Q84.1- Q84.6, Q84.8*
Chromosomal	758.0-758.8<br>*Exclusion:758.4*	Q90.0-Q99.2<br>*Exclusions: Q95.0, Q95.1*
Other	758.9, 759.0-759.9<br>*Exclusion: 759.9*	Q85.0-Q89.9, Q99.8, Q99.9<br>*Exclusion: Q89.9*
