# Supplementary figures and images for: The Canadian Mother-Child Cohort Active Surveillance Initiative (CAMCCO): Comparisons between Quebec, Manitoba, Saskatchewan, and Alberta
Source: PLoS One. 2022 Sep 20;17(9):e0274355. doi: 10.1371/journal.pone.0274355 (PMC9488808; doi:10.1371/journal.pone.0274355)

**S1 Fig. CAMCCO Infrastructure.**

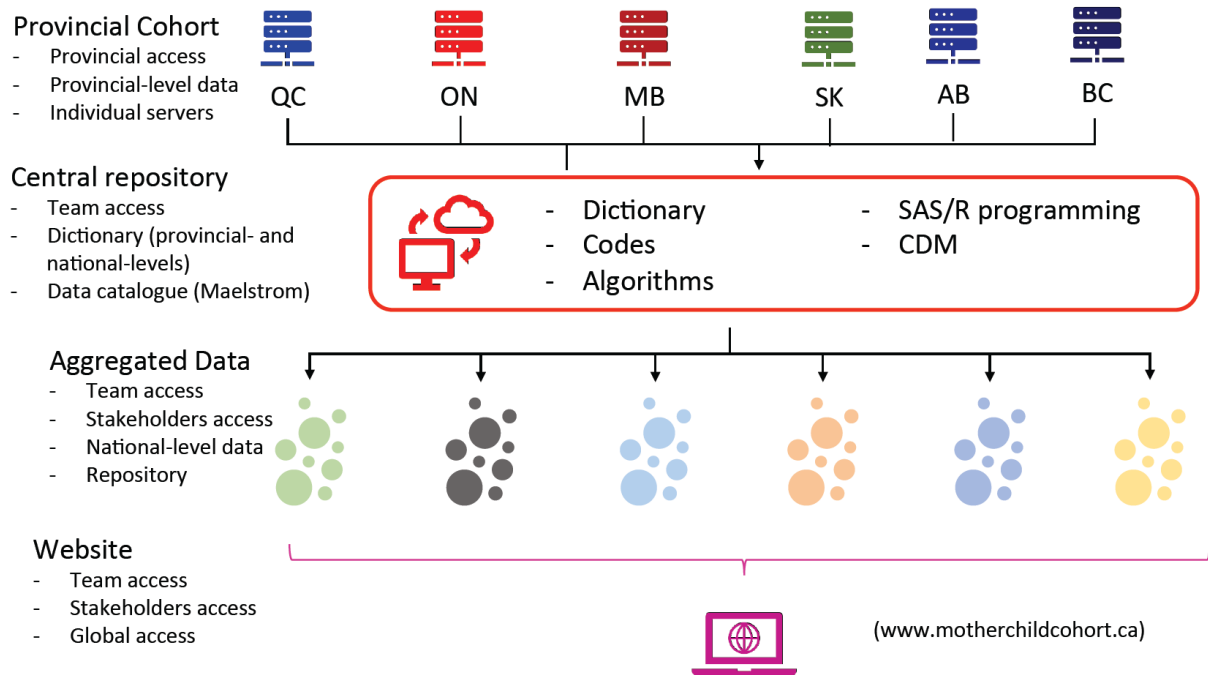

Supplement: S1 Fig — (PDF) [file pone.0274355.s003.pdf]

**S2 Fig. CAMCCO – Linkage procedures within each province.**

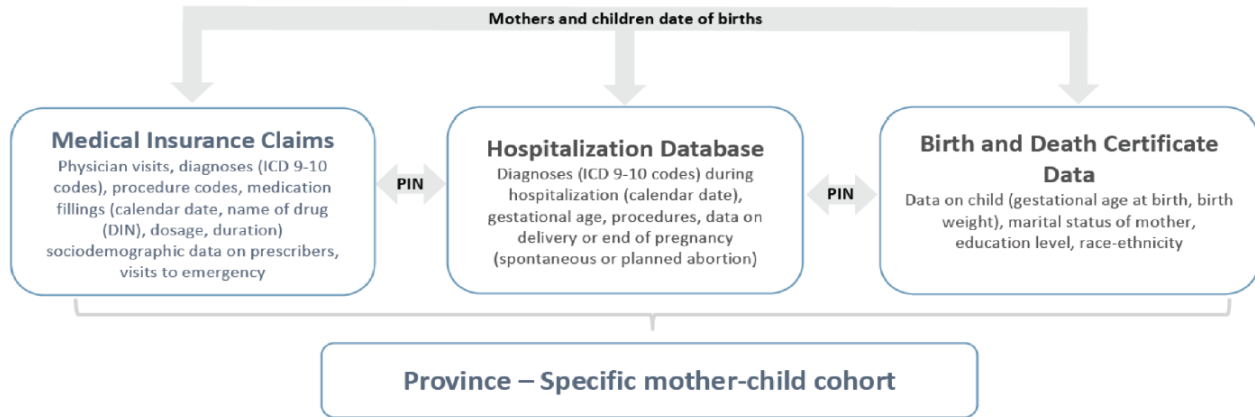

Supplement: S2 Fig — ICD 9–10: International Classification of Diseases 9th and 10th Revisions; DIN: Drug Identification Number; PIN: Unique Personal Identification number for mothers and children within each province. (PDF) [file pone.0274355.s004.pdf]

**S3 Fig. CAMCCO – Provincial cohort structure.**

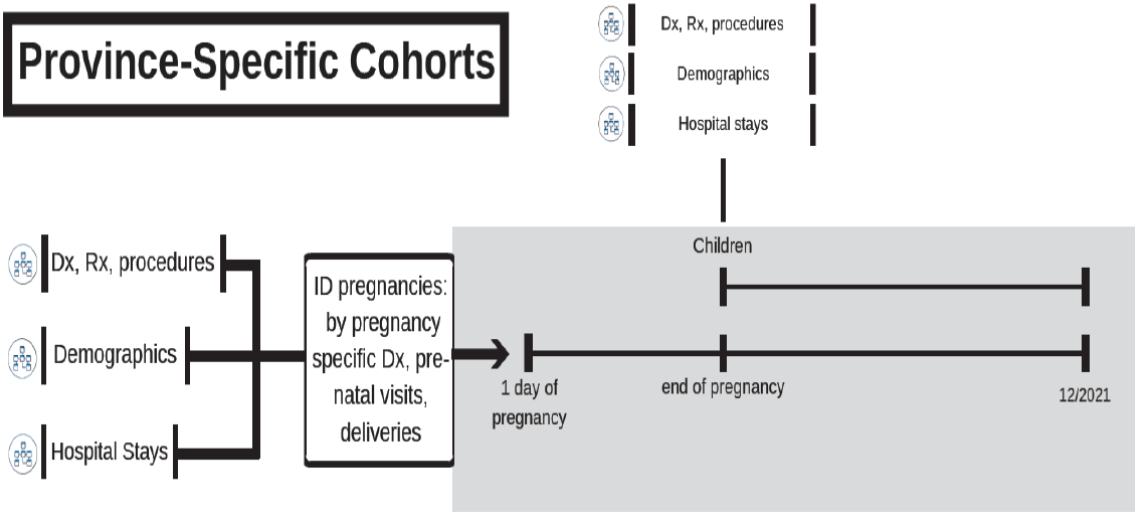

Supplement: S3 Fig — (PDF) [file pone.0274355.s005.pdf]

**S4 Fig. CAMCCO – Quebec, Manitoba, Saskatchewan, and Alberta – calendar years included in this study.**

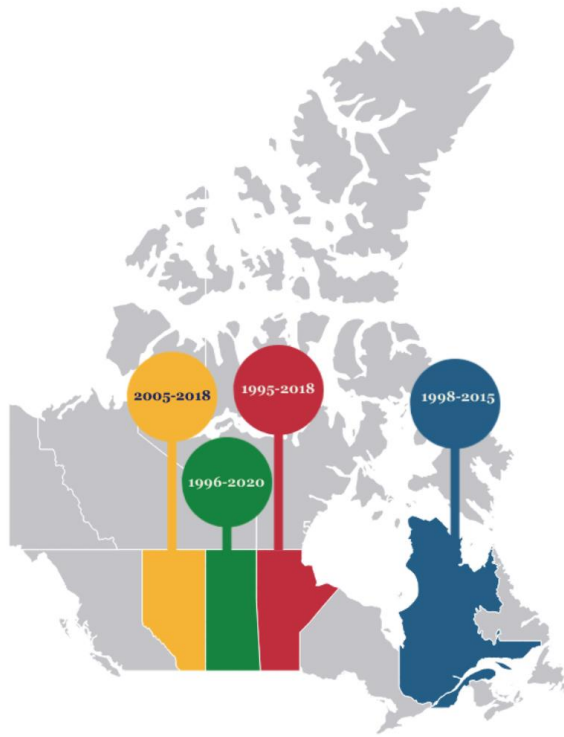

Supplement: S4 Fig — (PDF) [file pone.0274355.s006.pdf]
