# Supplementary material for: The Canadian Mother-Child Cohort Active Surveillance Initiative (CAMCCO): Comparisons between Quebec, Manitoba, Saskatchewan, and Alberta
Source: PLoS One. 2022 Sep 20;17(9):e0274355. doi: 10.1371/journal.pone.0274355 (PMC9488808; doi:10.1371/journal.pone.0274355)

S5 Fig. CAMCCO – Quebec, Manitoba, Saskatchewan, and Alberta – Overall prevalence of prematurity, LBW, and multiplicity.

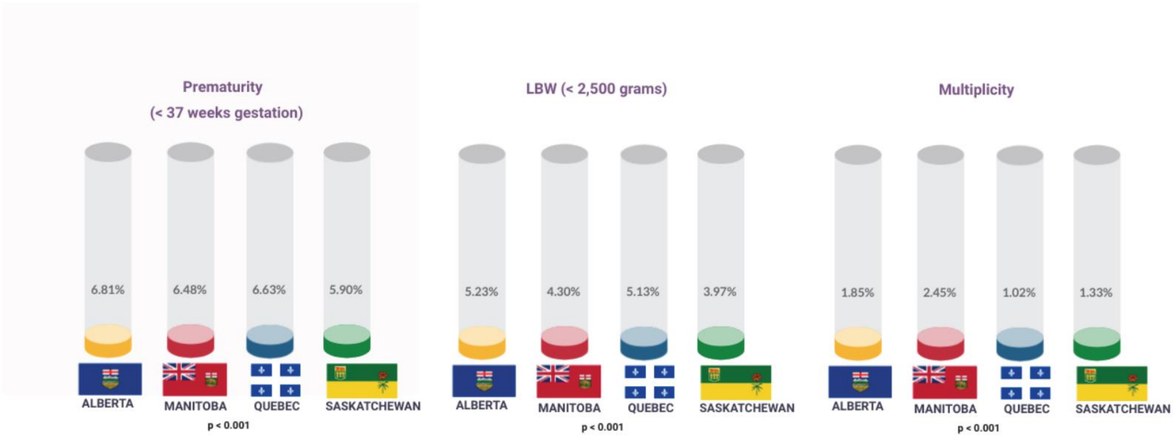

Supplement: S5 Fig — (PDF) [file pone.0274355.s007.pdf]
