# Supplementary material for: The Canadian Mother-Child Cohort Active Surveillance Initiative (CAMCCO): Comparisons between Quebec, Manitoba, Saskatchewan, and Alberta
Source: PLoS One. 2022 Sep 20;17(9):e0274355. doi: 10.1371/journal.pone.0274355 (PMC9488808; doi:10.1371/journal.pone.0274355)

S6 Fig. **CAMCCO - Quebec, Manitoba, Saskatchewan, and Alberta – Prevalence of specific malformations in the first year of life.**

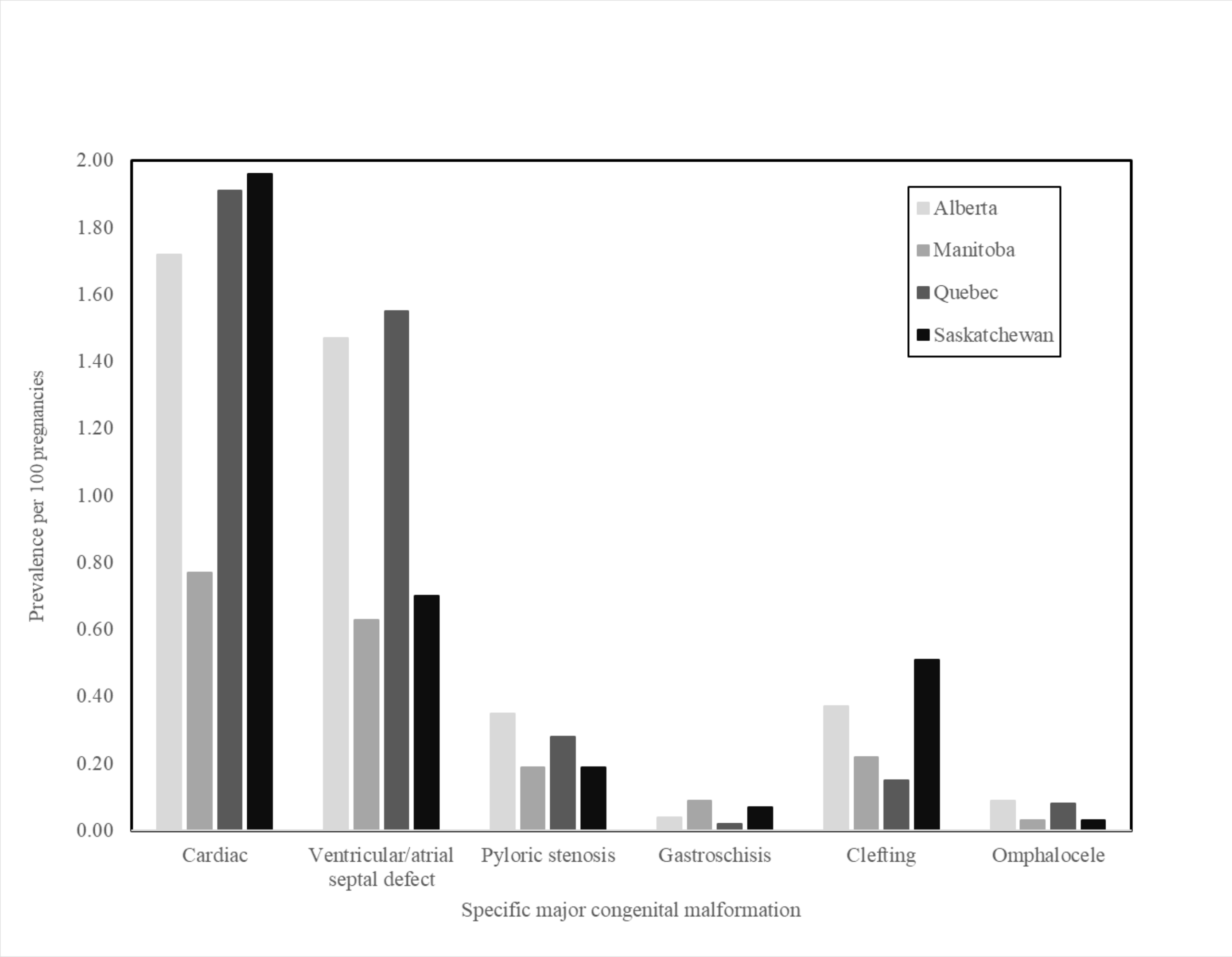

Supplement: S6 Fig — Note: All specific major malformation grouping prevalences between provinces were statistically significant (P<0.0001). (PDF) [file pone.0274355.s008.pdf]
